# Supplementary material for: Challenging Future Generations: A Qualitative Study of Students’ Attitudes toward the Transition to Animal-Free Innovations in Education and Research
Source: Animals (Basel). 2023 Jan 24;13(3):394. doi: 10.3390/ani13030394 (PMC9913108; doi:10.3390/ani13030394)
Supplement: Supplementary file 1 [file animals-13-00394-s001.zip › animals-2148750-supplementary.pdf]

CHALLENGING FUTURE GENERATIONS

Replacing Animal Testing Challenge

Course Guide

Academic Year 2021-2022

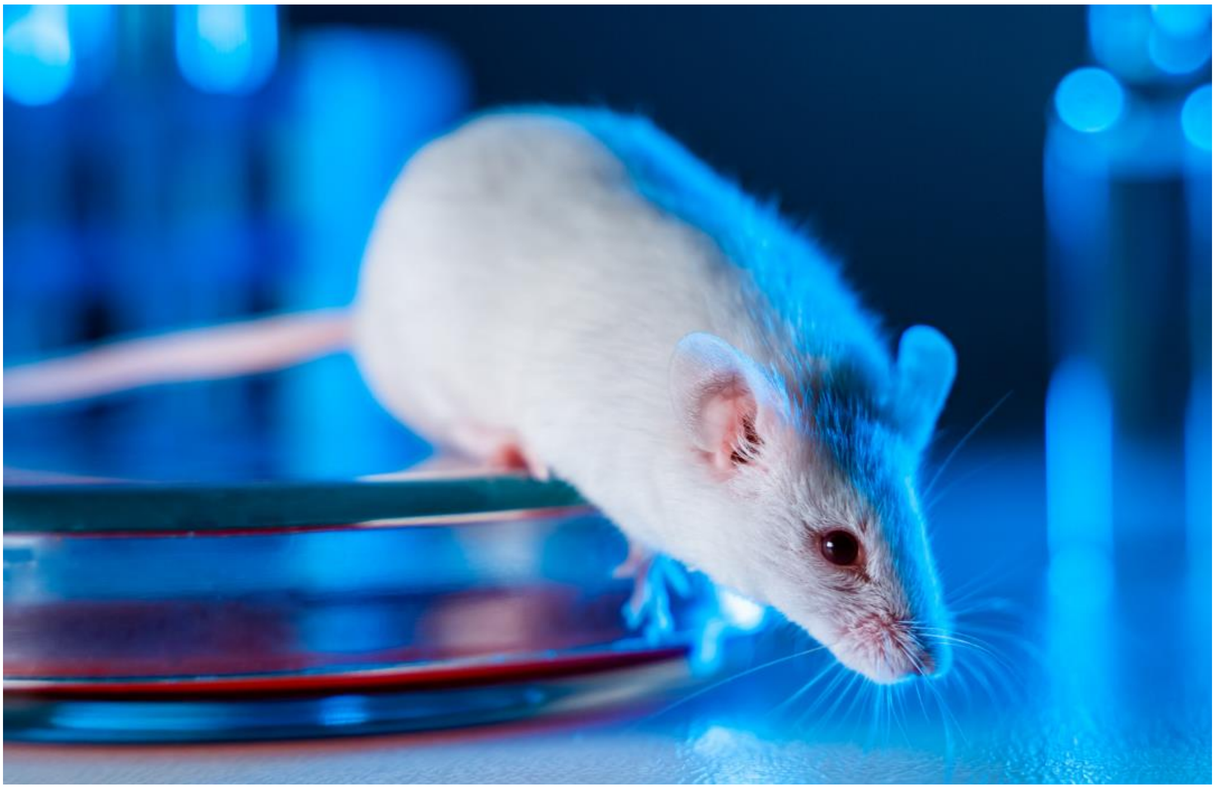

Table of content

General information 2
Introduction 3
Introduction to the challenge 3
Introduction to CBL 3
Learning outcomes 4
Roles and functions 6
Challenge roles 6
Team roles 7
Work load 7
Course Materials 7
Planning and schedule 8
Assessment 10
Individual Track 10
Team Track 10
Appendix 11
Template Advisory report 11
Rubrics for individual and group work 12

General information

**Course name:**	Replacing Animal Testing Challenge
**Credits:**	7,5 ECTS
**Open for:**	Third year BSc, MSc, PhD, Professionals and HU students
**Period:**	14th of February 2022 – 1st of July 2022
**Course Coordinator:**	Daniela Salvatori (UU)
**Challenge agent:**	Debby Weijers (Proefdiervrij)
**Course Examiners:**	Daniela Salvatori (UU), Ludo Hellebrekers (WUR), Karin Peters (WUR)
**Contact person UU:**	Daniela Salvatori (d.salvatori@uu.nl)
**Contact person TU/e:**	Erik van der Spek (e.d.v.d.spek@tue.nl)
**Contact person WUR:**	Ludo Hellebrekers (ludo.hellebrekers@wur.nl)
**Contact person HU:**	Ronald Vlasblom (ronald.vlasblom@hu.nl)<br>Rinske Drost (rinske.drost@hu.nl)

Introduction

Introduction to the challenge

One of the main goals behind this course is to offer you the opportunity to work on a real world problem, applying your interdisciplinary knowledge coming from the various (academic) backgrounds you have as well as personal/cultural, in order to provide a solution to the challenge. So what is the challenge about?

Every year, 10 million animals are used for scientific purposes. A similar number of laboratory animals are bred every year for science but never actually used. Are these animals necessary, or is it time for modern science to make a change? If we stop animal testing today, what would the world look like?

When we buy food or drinks in the supermarket, we can assume that these products are not harmful to our health. If you take a look around your kitchen, there is a big chance that all the food and drinks you find have at some point been tested on animals. Food safety research not only tests the final product but looks at the whole production process. Ingredients, chemicals, flavouring, packaging materials and the machines used to produce the food or drinks: simply everything to do with food has been tested on animals. Is there a way to change this situation?

Next to food safety control, animal testing is also mandatory for vaccine batch control. The Netherlands has embraced the objective of striving to phase out the regulatory required animal testing, but vaccine batch control involving animal testing is still mandatory for some types of vaccines. To phase out animal testing, animal-free methods must be developed. What have we learned from situations such as the covid pandemic? What are the hurdles? What are the opportunities? How should industry, research institutions and control authorities collaborate in order to introduce animal-free methods?

Problems in using animals are both practical and fundamental. To date, tests on animals are central to human medicine and food safety. During the first steps of drug development and toxicity screening, animals are used to identify promising and safe compounds, but the obtained results are hard to translate to the human situation. After all, animals are not humans. As these substances are further refined, they undergo a variety of animal tests. In the final step before research in humans, animals are used in toxicity testing to determine whether these drugs show an appropriate balance of safety and efficacy. Despite this systematic use of animals in the various stages of preclinical research, 95% of the pharmaceuticals that show promise in preclinical animal research and proceed to human clinical trials however fail to make it to market and to patients waiting for a cure.

Both fundamental and practical problems related to the use of animals for producing medicine are heavily discussed by the industry, academia, and the government. It is particularly challenging for biopharmaceutical companies and start-ups to overcome the hurdle between research and actual release of new therapies and food compounds. This hurdle is called the “valley of death” for multiple reasons. Can we innovate this complex ecosystem of medicine production (large companies, start-ups, universities, research centres) and cross the “valley of death” without using animals?

To accomplish this, we must start focusing on educating the next generation of researchers and educators. That is why you are challenged to find better ways to perform research in healthcare and food safety testing. Together with a team, you will find an answer to what our world would look like if lab animals were obsolete.

For 20 weeks, you are going to collaborate in mixed teams of students from TU/e, WUR, UU and UMCU and receiving input from researchers from all these institutes as well as from Proefdiervrij. At the end of the Challenge, each team needs to deliver an advisory report and a proof of concept that validates your idea and a pitch effectively communicating your concept to the client and a jury. Apart from this, you will be allocating part of your course hours to develop your personal learning goals. In the following chapters you’ll find out more about this challenge, its learning outcomes, deliverables and assessment.

Introduction to CBL

The RAT Challenge is a Challenge Based Learning (CBL) course. The way this challenge is organized might be different then you're used to. In this challenge you're in control of your own learning and process. You have the responsibility to take initiative and make decisions. On the one hand on team level and on the other on individual level. Furthermore, we work with a challenge agent (Proefdiervrij) who is outside the academic world. Proefdiervrij is your first important stakeholder. Debby from Proefdiervrij is offering you a highly complex problem: replacing animal testing. There is no one-size-fits all solution. So, this challenge will lead to several solutions applicable for different stakeholders and end users. We guarantee that you'll be faced with uncertainties and knowledge gaps. We'll not offer you a course schedule with all the details set in stone. We offer you a foundation at least in the first 5 to 8 weeks, but you're in charge and responsible to explore the knowledge domains and expertise within your team and to define the knowledge gaps. It's up to you to involve stakeholders and include different types of knowing (academic and non-academic), perceptions and experiences to develop a feasible and viable solution. It is your investigation, your project, your challenge. To help you get started, we'll offer you several tools and workshops you can use in this process. For instance, we

start with an interdisciplinarity workshop at the kick-off event and you'll be supported by a coach. Even though every team will work on their own solution, you might need to consult each other once in a while as well. So know that if your team doesn't have an answer, maybe another might. In short, you're cocreating this challenge. We will invite you to tap into your knowledge, personality traits, talents, interests, experiences and competencies to learn with each other and from each other. And we're looking forward to be learning from you.

Learning outcomes

In this chapter the Learning Outcomes (LOs) are listed. These LOs are expected to be developed by you during this course. You will notice that the first 4 LOs are mainly focusing on the team part and the last LO is focusing on the individual part.

After successful completion of this course students are expected to be able to:

1. Analyse the context of a real-life problem and distinguish the most relevant elements for designing a solution;
2. Assess opportunities offered by the existing state-of-the-art technologies, methods and solutions and identify areas for innovation, relevant for solving a real-life challenge;
3. Gathering, selecting and analyzing information, tools and techniques and integrating this into final project deliverables in terms of process and content at an academic level;
4. Discuss and defend their viewpoints and conclusions in a professional and academically correct way;
5. Set and reflect on personal learning goals based on their expertise and that of other members and stakeholder, in a cyclic manner leading to future reflections and actions.

In table 1 an overview is given of these learning outcomes, the indicators that are identified to assess these outcomes and the ways in which the learning outcomes are assessed.

Table 1 Overview of learning outcomes

Learning Outcomes	Indicators	How/what assessed?	Assessed by whom?
**1. Analyse the context of a real-life problem and distinguish the most relevant elements for designing a solution;**	- Analysis of the identified issues and their interrelations.<br>- Extent to which description of current situation is based upon relevant information backed by data or references.<br>- Presence of critical reflection on the methods and results.	Group level<br><br>Advisory Report<br><br>Feedback by coach, experts and challenge agent	Final assessment by assessment committee
**2. Assess opportunities offered by the existing state-of-the-art technologies and solutions and identify areas for innovation, relevant for solving a real-life challenge**	- Extent to which analysis is supported with arguments and evidence.<br>- Engagement with the solutions presented.	Group level<br><br>Advisory Report<br>Proof of Concept<br><br>Feedback by coach, experts and challenge agent	Final assessment by assessment committee
**3. Gathering, selecting and analysing information, tools and techniques and integrating this into final project deliverables in terms of process and content at an academic level**	- Evaluation of the methodology and design approach in terms of clarity, conciseness, completeness and factual errors, contradiction or omissions.<br>- Deliverables aligned with real life problem/commissioner.	Group level<br><br>Advisory Report<br>Proof of Concept<br><br>Feedback by coach, experts and challenge agent	Final assessment by assessment committee

**4. Discuss and defend their viewpoints and conclusions in a professional and academically correct way;**	- Evaluation of recommendations and conclusions in terms of clarity, concreteness and applicability.<br>- Conclusions follow logically from the findings of the study.<br>- Extent to which oral presentations are enthusiastic (audience's attention) and purpose of the presentation is clear.	Group level<br><br>Advisory Report<br><br>Feedback by coach, experts and challenge agent	Final assessment by assessment committee
**5. Set and reflect on personal learning goals based on his/her expertise and that of other members and stakeholder, in a cyclic manner leading to future reflections and actions.**	- Formulation of learning goals in terms of I, WE and IT (personal, clear, explicit).<br>- Description of an action plan that will lead to achieving learning goals in a cyclic manner.<br>- Ways in which is described if/how learning goals are achieved (incl. actions that have been taken).<br>- Use of feedback and/or scientific literature.	Individual level<br><br>Feedback by coach, peers and experts	Final assessment by assessment committee based on grading by coaches

Roles and functions

Challenge roles

Course coordinator

The course coordinator is responsible for organisation of the course and can answer questions about subjects that have not been specifically assigned to other staff members as indicated below. The course coordinator is also available when organisational issues are unclear, problems with your coach occur, when a student does not function in a team or when a problem with the commissioner cannot be solved by the team and the coach. Finally, the course coordinator can be contacted in case of questions on the final assessment.

Course examiners

This course consists of two parts that will be assessed separately, the individual and group part. The course examiners are determining the final grade for each student. There are three examiners, one of which is the course coordinator. For the team product the course examiners will support their decision on the input of the jury and the challenge agent. For the individual part the course examiners will receive the grades for each student from the coaches. The examiner may revise the mark after consulting the coaches. If a student has an insufficient grade, then the examiners will decide whether the student has to re-do the course, or assign additional work in order to complete it.

Challenge agent

The challenge is introduced by the challenge agent, Proefdiervrij. They will have an advisory role in the assessment. The challenge agent will be present during at least the kick-off and final event. The challenge agent supports the process of formative assessment. At different phases of this challenge, student teams share and pitch their work in order to receive feedback from an industry perspective.

Coach

During this course you and your group will be assisted by a coach. Every team has its own coach, who will also be the coach of the individuals that are in that team. The coach provides feedback on your learning goals and guides the process of your team. Set feedback moments with the coach are planned throughout the challenge, but students are free to their coach for extra feedback. The coach supports and challenges the development of you and your team, helps you to (learn to) reflect on where you are at each point in the process in regard to the competence and professional skills development, where you would like to and how to get there. So the coach is not there to help them directly in the project, but to help them in the process. Your coach is also added in your MS teams - team channel so you can always reach out to him/her and arrange meetings/ chats throughout the challenge.

You can expect the following:

- The coach will organize the first team coaching meeting. In most cases this will take place during the kick-off event..
- The coach will perform at least four individual meetings with each team member: a starting interview, two midterm performance meetings (where the student and the coach discuss the learning experience) and a final performance interview (where the student presents their own learning experience).
- The coach is available for all questions or issues related to the team functioning
- • Be aware that your coach is not necessarily an expert in the fields relevant to your project. If the coach is also an expert make consultation appointments next to coaching appointments to avoid a mix up of process coaching and content advising.
- Being present during a meeting does not imply that the coach will tell you what to do. The coach will also observe, make notes and can discuss the observations after the meeting to help the team with self-reflection and functioning.

Experts

During the course several experts on specific fields are involved in the challenge offering the teams different kinds of knowledge and insights and helping them in defining the problem definition and working on their solution. An overview of experts will be posted on MS Teams and is expected to be updated throughout the course since more experts might be joining still. In the first 5 weeks, expert sessions are scheduled, from week 6 onwards the expert sessions will be scheduled upon request by the student teams. Experts can still be contacted on student teams' initiative throughout the challenge. Experts will be present during the events,

facilitating expert sessions, join a Q&A, and/or join as jury members for your final presentation. The challenge agent is also part of the expert "pool".

Students

Students are asked to work with other students and experts to acquire knowledge by engaging in this real-life interdisciplinary challenge. While working on prototype solutions that contribute to this challenge, students seek out and apply knowledge, individually or in groups. The students are responsible for their own learning and prepare meetings with experts and their coach.

Team roles

The most important role of each team member is to provide disciplinary input into the work for the proposal and later on the products of the team and learn new disciplinary knowledge throughout the challenge. Other than that, assigned team members should be in charge of the team function in terms of staying on track, following the planned activities, revising plans if needed and communicating properly with the stakeholders. During meetings someone will have to be the assigned as chair and someone will have to be assigned as reporter. These functions may rotate as all students are expected to be able to carry out these tasks. To make teams perform more professionally though, the following team roles can be assigned to individual students.

Project manager: The project manager is responsible for the general coordination and functioning of the team, for defining the project goals, for the contacts with the challenge agent and other stakeholders. The project manager will usually chair the team meetings with the challenge agent. Specific skills: communication and interpersonal skills. Management and decision-making skills.

Secretary: The secretary is responsible for the planning and preparation of team meetings (together with the project manager) and sees to it that minutes of meetings are available and that decisions have a follow-up. The secretary is also responsible for administration, correspondence and public relations. Specific skills: communication skills, organisational skills.

Controller: The controller is responsible for planning, budgeting (every team has a small budget available for prototyping) and controlling of both the team work and the project. Specific skills: Skills in financial book keeping and planning. He/she is able to keep track of the project's actual and planned progress and persuade team members to deliver or the team to revise the planning. Specific skills: organisational skills, experience with Excel.

Project member: Where the former functions are mainly management related, the team members are the ones who should guard the quality of the work so this is the more content related function. Together with the other group members they are responsible for both general and specific tasks, like exploring available sources of information and the correct selection and analysis of relevant data (e.g. from libraries and the internet). Specific skills: Good assessment ability regarding the quality and relevance of information. Experience with analysing and presentation techniques (e.g. SAS, Genstat, Excel, PowerPoint, and GIS packages).

Work load

The student is expected to work 10h a week on this challenge which includes the groupwork and working on the individual track. Coach sessions and events are also included in these 10 hours.

Course Materials

All the activities will be facilitated via MS Teams. Each team will have its own channel and can also make use of the features provided. You can make use of the common folder of documents for collaborations etcetera. During the challenge you will make use of MS teams where we share content, recordings and updates and provide you with feedback.

If you are not yet familiar with how to use MS teams you can find an elaborate description of the video software at this link.

Planning and schedule

During 20 weeks you will work together in a multidisciplinary team to create a proof of concept and an advisory report. The Challenge will start the 14th of February with an official opening on the 16th during a kick-off meeting, where you meet your team and coach with whom you will work together. During the kick-off meeting you'll be working on a first direction of your problem definition therefor in preparation we'd like to ask you to watch the knowledge clips of the first week. After the kick-off you will as a team continue working on your problem definition which you can ask for feedback in week 3 or 4 by the challenge agent. You will at least get feedback during the 2nd event when you will pitch your problem definition. After the problem definition, you will continue working on your first ideas for a solution.

Hereby the schedule of the first 5 weeks. More details will follow during the challenge.

Date	Activity	Location
	Week 1	
7th February – 16th February	Challenge preparation e.g. knowledge clips and introductory documents	MS teams
**16th of February whole day**	**EVENT 1: Kick-off**	**UU Anatomiegebouw**
	Self-study on individual learning goals	MS teams
	Week 2	
**23rd of February**<br>- **12:30h-13:30h**<br>- **16:00h-17:00h**	Expert sessions 1 & 2	MS teams
**25th of February 12:30h-13:30h**	Expert session 3	MS teams
	Consultation by Challenge agent on problem definition, team work and self-study (knowledge clips, online tools)	MS teams
	Week 3	
**2nd of March**<br>- **12:30h-14:00h**<br>- **16:00h-17:00h**	Workshop exploring problem definition techniques & Expert session 4	MS teams
**4th of March 16:00h-17:00h**	Expert session 5	MS teams
	Team work and self-study (knowledge clips, online tools)	MS teams
	Week 4	
**8th of March**<br>- **12:30h-13:30h**<br>- **16:00h-17:00h**	Expert session 6 & 7	
**11th of March**<br>- **12:30h-13:30h**<br>- **16:00h-17:00h**	Stakeholder mapping & Expert session 8	
	Team work and self-study (knowledge clips, online tools)	MS teams
	Week 5	
**11th of March 12:30h-13:30h**	Expert session 9	
**18th of March whole day**	**EVENT 2: Pitching problem definition and first ideas**	tba
	Week 6 - 20	
**11th of May whole day**	**EVENT 3: Towards a product**	**tba**
**1st of July whole day**	**EVENT 4: Grand final**	**tba**

Throughout the weeks we will schedule expert sessions and workshops especially until week 8, after that there will be more emphasis on groupwork. The following timeslots are reserved for expert sessions: **Wednesdays and Fridays: 12:30h-13:30h & 16:00h-17:00h.** We will not fill these time slots every week, but if there are

sessions, they will take place on these timeslots. If you are unable to attend an expert session, you will need to discuss with your team who can attend. For the workshops we strongly advice you all to attend individually. A more elaborated schedule will be provided on MS teams.

When?	Feedback on	Form of feedback	Whom?	How?
**Between event 1 and 2**	Individual learning goals	Reviewing content on learning goals, refining portfolio/meeting	Coach<br>Students	Students request, online
**Between event 1 and 2**	Project proposal		Coach<br>Experts<br>Challenge agent	Teams request, online
**Event 2**	Feedback on teams' problem definition and first ideas	Peer feedback on pitch and problem definition	Experts<br>Teams<br>Challenge agent	Meetings during event
	Individual learning goals	Request feedback on personal learning goals (team or individual activity with the coach)	Coach<br>Students	Student request, during event
**Between event 2 and 3**	First draft proof of concept	feedback on proof of concept	Experts<br>Challenge agent	Teams request, online
**Event 3**	Feedback on proof of concept	Peer feedback on pitch and concept	Coach<br>Team	During event 3
	Individual learning goals	(peer) feedback on personal learning goals (team and/or individual activity with the coach)	Coach<br>Students	Students request[1]
	Feedback on Proof of Concept	Short pitch on proof of concept, Q&A session and feedback.	Challenge agent<br>Experts	During event - experts visit teams
**Between event 3 and 4**	Q&A with expert group members.	Feedback on team process and products (report, proof of concept)	Experts<br>Challenge agent	Teams request
	Individual learning goals	Request (peer) feedback on personal learning goals (team and or individual activity with the coach)	Coach	Students request
	Proof of concept and advisory report	Jury receive a week in advance of the final event these documents	Jury	Online
**Event 4**	Team meeting: preparation grand pitch/portfolio	Team meeting with coach to practice pitch and receive feedback on final concept	Coach	During event
		Peer feedback pitch and concept (team/team)	Teams	During event
	Individual goals and portfolio	Individual meetings with coach on portfolio and learning goals	Coach	During event

[1] Student must meet with the coach at least two times between the kick-off meeting and the exit meeting with the coach

Assessment

During the challenge, different forms of assessment will be applied. Every student will be assessed based on an individual track and a team track. The individual track will account for 30% of the final grade, whereas the team track will provide 70% of the final grade. For both individual and group work, rubrics are available. Grading is based on these rubrics.

Individual Track

In the individual track, you will set your own learning goals with the support from a coach. You decide when to collect evidence to support your progress, as well as when to have feedback moments. During the course there are in total 2h allocated to have four individual feedback sessions with your coach. These sessions focus on supporting and guiding you throughout the learning process that suits your learning goals. The first session is focused on defining the learning goals. The following two are for feedback and update and the last session is a check out point. When you need more sessions, this can be discussed with the coach. The first session as well as the check-out are set and scheduled during the face to face events. The sessions in between will be planned by you, based on personal learning needs, therefore all students will plan these meetings with the coach during the first individual coaching session. In an online portfolio, every student collects written (peer)feedback and adds reflections. These reflections are input for meetings with the coach. The coach will moderate the reflection cycle so students will experience growth based on new insights from these reflections. During these meetings, you are in charge of the topic to be discussed. The coach will provide feedback, if asked, and then go into a discussion and exploration of the next steps in order for the student to reach each final learning goal.

Team Track

All student teams are asked to deliver a solution to the challenge provided by Proefdiervrij. This solution should be sustainable, suitable and satisfactory to the user, as well as aligned with the expectations of the challenge agent for an innovative and feasible concept. Each student team will form its own concept, after receiving input from experts and interviewing the challenge agent and other stakeholders at their own initiative.

At the beginning of the course, teams prepare a project proposal, of which a first draft is discussed with the coach on process. Project proposals ideally address the aim of the project and research questions, the planning chart, the stakeholder analysis, go/no-go decisions and involved risks. The proposal can be presented and provided with feedback by the challenge agent and the experts. During project execution it is advised that the team checks the project proposal and negotiates adjustments, also with the challenge agent, when and if needed.

Whichever path the team follow, by the end of the course all teams need to deliver two main products, a **proof of concept** and an **advisory report**. These products will need to be delivered to the challenge agent and the assessors a week in advance (24th of June '22) from the final event (1st of July '22) of the challenge in MSteams.

1. Proof of concept

The aim of a 'proof of concept' is to demonstrate the feasibility of the proposed solution. A proof of concept is a visual demonstration of the idea, proving to the challenge agent the feasibility, scalability and practical potential of the concept by suitable means. It could be in a form of a physical prototype, simulation, video, power point, movie, model, etc.

2. Advisory report

The advisory report is a document that includes the research process and results, as well as an elaborate description of the solution, its properties and implementation plan. This report is a product that the accessors can use in order to access whether the solution that is suggested is innovative, feasible, viable and desirable for the user. Concerning the format of an advisory report, we expect a document of 10 A4 pages that will include certain basic points and have specific elements (see Appendix 1). The students should keep in mind certain information that the challenge agent wishes to receive from the report such as: problem definition (what is the challenge), target group, existing solutions or technologies that would be an opportunity, argumentation on the chosen solution based on the problem and research that took place, potential of further development of the current solution (relate it to developing/future technology), include a financial analysis, SWOT analysis, implementation plan and parties that will be involved, as well as the proof of concept description and validation process.

Appendix

Template Advisory report

The structure of an advisory report can be as follows:

- *Title Page:*
  Include Title/subtitle, author(s) name(s), challenge agent(s), place, date and year of writing (1A4 page)
- *Executive Summary:*
  Provide a summary of your suggestion (and justification) to the challenge agent (max 1A4 page)
- *Table of contents:*
  (1A4 page)
- *Introduction:*
  Introduce the problem given to you, including background and context. Moreover include in this section the main question that was explored, focus group and study's parameters. (max 1A4 page)
- *Research design*
  This section includes your research design (2 A4).
- *Alternative measures/solutions:*
  This section outlines possible steps and then weighs them against constraints that might have been faced during the process. Using the research that was performed as a foundation, each measure is substantiated (max 4A4).
  The following questions could function as a guideline:
    - How would the measure contribute to solving the problem and in how much time?
    - What are the measure's advantages and disadvantages?
    - Is there evidence that demonstrates that the measure would work?
    - What would implementing this measure require in terms of money, time, people and the organization itself?
- *Conclusion:*
  The conclusion identifies your ultimate recommendation and explains the justification of your solution (max 1A4). It should provide the following:
    - The solution you are suggesting
    - The steps the challenge agent needs to take and in which order
- *Sources*

Rubrics for individual and group work

**Individual work**					
	**insufficient**	**sufficient**	**good**	**excellent**	**weight**
**Learning goals**	Learning goals are impersonal (e.g. copied from the course goals), vague, unclear or non-existent.	Learning goals are fairly well-defined and personal, but are "safe", i.e. can easily be achieved just by attending the<br>course. They do not encompass '*und erstanding'*,<br>*'organizing'* and *'influencing'.*	Learning goals are clearly-defined, personal and ambitious, i.e. the student has to put in extra effort or move out of his/her comfort zone to achieve them. They encompass *'understanding', 'organizing'* and *'influencing'*.		10%
**Action plan**	Action plan and baseline measurement are almost absent or merely descriptive	Effort was made to do a baseline measurement. Action plan is described clearly, with some specific examples, there is an attempt to do this in a cyclic manner, but it may be superficial.	Baseline measurement is made. Action plan is described clearly and concisely, with some specific examples and not always in a cyclic manner.	Good effort to make a baseline measurement and action plan is described clearly and concisely, with specific examples and in a cyclic manner.	30%
**Reflection report**	Achievement of learning outcomes is absent or merely described, with little attempt at reflection.	Achievement of learning outcomes is described clearly, with some specific examples. There is a clear attempt at reflection, but it may be superficial or only cover some of the elements requested (knowledge, contribution to the problem, interdisciplinarity, influence of values/interests).	Achievement of learning outcomes is described clearly and concisely, with specific examples. Reflection is adequate and covers most of the elements requested (knowledge, contribution to the problem, interdisciplinarity, influence of values/interests).	Achievement of learning outcomes is described clearly and concisely, with specific examples. Reflection is excellent and covers all the elements requested (knowledge, contribution to the problem, interdisciplinarity, influence of values/interests). It is clear what the student has learnt from the project and how s/he can use this in future projects.	50%
**External resources**	Feedback and scientific literature are missing or so poor that they are useless.	Is able to show how the use of feedback and scientific literature supported the learning process.	Is able to show how the use of feedback and scientific literature supported the learning process and led to new insights and connections.		10%

Group Work				
	**insufficient**	**sufficient**	**good**	**excellent**
**Challenge analysis**	Data is not analysed, at most it is described.	A fair attempt at analysis from an inter-disciplinary perspective. Themes are identified. Observations, surveys and interviews are somehow triangulated. Statistical methods are mostly clear and appropriate. Arguments are generally backed by data or literature.	A good analysis from an inter-disciplinary perspective. Themes are clearly described. Observations, surveys and interviews are well triangulated. Statistical methods are clear and appropriate. Arguments are generally backed by data or literature.	An excellent analysis, with good use of data, evidence of extensive further reading and original thinking. Statistical methods are clear and appropriate. Arguments are backed by data or literature. Taking into account a trans-disciplinary perspective.
**Description current situation**	Current situation is not described or so incompletely that it is useless.	Current situation is described but some information is irrelevant and assertions are frequently not backed by data or references. Description may be unclear, incomplete, contain factual errors or contradictions. Treatment of the different topics (expert analyses) is very unequal for no apparent reason.	Current situation is described satisfactory, the information is mostly relevant and assertions are generally backed by data or references, but there is little critical reflection on the methods and results. Description is clear, but may contain some factual errors or contradictions. Treatment of the different topics (expert analyses) is balanced or explanations are given if not.	Excellent description of current situation, including relevant information backed by data or references, with critical reflection on the methods and results. Few (if any) factual errors or contradictions. Treatment of the different topics (expert analyses) is balanced or explanations are given if not.
**Methods & Results**	Results or methods are presented without reflection.	Results are presented with some reflection of data or methods.	Both methods and results are critically reflected upon.	Excellent discussion on both the methods used and the results found leading to good insight of the value and limitations.
**Analysis of opportunities**	Presents an incomplete analysis of some of the opportunities with no or limited arguments and evidence.	Presents an inter-disciplinary, but superficial, analysis of some of the opportunities with some arguments and evidence.	Presents a thorough inter-disciplinary analysis of most opportunities with sufficient arguments and evidence.	Presents an insightful, thorough trans-disciplinary analysis of all opportunities and their interrelations.
**Proposed solution**	No action suggested and inappropriate solutions proposed to the issues in the project.	Little action suggested and/or inappropriate solutions proposed to the issues in the project.	Support diagnosis and opinions with limited reasoning and evidence; presents a somewhat one-sided argument; demonstrates little engagement with the solutions presented.	Support diagnosis and opinions with strong arguments and well documented evidence; presents a balanced and critical view; demonstrates engagement with the solutions presented.

**Methodology and design**	Methodology and/or design is missing or so incomplete that it is useless.	Methodology and design approach contains many factual errors, contradictions or omissions. It does not link the methods used or design approach to research aims.	Methodology and design approach is mostly complete and free of factual errors, contradiction or omissions. It links the methods used and the design approach to the research aims.	Methodology and design approach is clear, concise, complete and free of factual errors, contradiction or omissions. It links the methods used and the design approach to research aims and explains why these methods are appropriate.
**Proof of concept**	No attempt is made to demonstrate the feasibility of the proposed solution.	Suitable means are selected to demonstrate the feasibility of some elements of the proposed solution.	The means selected, to demonstrate the feasibility of the proposed solution, are supported by valid arguments.	Multiple iterations are made to demonstrate the feasibility of the proposed solution (via suitable means), each iteration is supported by valid arguments.
**Recommendations and conclusions**	Conclusions and/or recommendations are missing, unrealistic or bear no relation to the findings of the study.	Conclusions and/or recommendations are quite general or vaguely formulated. They may not be applicable in the short to medium term and do not always follow logically from the findings of the study.	Recommendations and conclusions are mostly clear, concrete, applicable in the short to medium term and follow logically from the findings of the study. There is some effort to draw on examples of best practice, but it may be unclear how these might be adapted to the local context.	Recommendations and conclusions are clear, concrete, applicable in the short to medium term and follow logically from the findings of the study. There is evidence of original thinking: new solutions or adaptation of best practice to the local context.
**Communication to stakeholders**	Unable to adapt their message to the appropriate level of elaboration to different stakeholders, orally and/ or in writing.	Able to adjust the level of elaboration in the verbal and written communication to the target audience, both during the project and in the presentation of the final results. In doing so at least three of the below elements could be improved; *the communication was: relevant, informative, kept the attention of the audience, had good command of the language and led to effective interactions.*	Able to adjust the level of elaboration in the verbal and written communication to the target audience, both during the project and in the presentation of the final results. In doing so one or two of the below elements could be improved; *the communication was: relevant, informative, kept the attention of the audience, had good command of the language and led to effective interactions.*	Able to adjust the level of elaboration in the verbal and written communication to the target audience, both during the project and in the presentation of the final results. In doing so *the communication was: relevant, informative, kept the attention of the audience, had good command of the language and led to effective interactions.*
